# Oxidative Stress as an Underlying Contributor in the Development of Chronic Complications in Diabetes Mellitus

**DOI:** 10.3390/ijms14023265

**Published:** 2013-02-05

**Authors:** Suziy de M. Bandeira, Lucas José S. da Fonseca, Glaucevane da S. Guedes, Luíza A. Rabelo, Marília O. F. Goulart, Sandra Mary L. Vasconcelos

**Affiliations:** 1 Medical Course of Cariri, Federal University of Ceará, Barbalha, Ceará 63180-970, Brazil; E-Mail: suziy@oi.com.br; 2 Northeast Network on Biotechnology (RENORBIO), Federal University of Alagoas (UFAL), Maceió, Alagoas 57072-900, Brazil; E-Mails: glaucevane@yahoo.com.br (G.S.G.); luizaa.rabelo@gmail.com (L.A.R.); mariliaofg@gmail.com (M.O.F.G.); 3 Laboratory of Cardiovascular Reactivity, Institute of Biological Sciences and Health, Federal University of Alagoas (LRC/ICBS/UFAL), Maceió, Alagoas 57072-900, Brazil; E-Mail: lucasjsdf@hotmail.com; 4 Faculty of Nutrition, Federal University of Alagoas (FANUT/UFAL), Maceió, Alagoas 57072-900, Brazil; 5 Institute of Chemistry and Biotechnology, Federal University of Alagoas (IQB/UFAL), Maceió, Alagoas 57072-900, Brazil; 6 National Institute for Science and Technology (INCT)–Bioanalysis, Campinas, São Paulo 13083-970, Brazil; 7 Laboratory of Nutrition in Cardiology and Comorbidities, Faculty of Nutrition, Federal University of Alagoas (NUTRICARDIO/FANUT/UFAL), Maceió, Alagoas 57072-960, Brazil

**Keywords:** biomarkers, diabetes mellitus, hyperglycemia, oxidative stress, reactive nitrogen species, reactive oxygen species

## Abstract

The high prevalence of diabetes mellitus and its increasing incidence worldwide, coupled with several complications observed in its carriers, have become a public health issue of great relevance. Chronic hyperglycemia is the main feature of such a disease, being considered the responsible for the establishment of micro and macrovascular complications observed in diabetes. Several efforts have been directed in order to better comprehend the pathophysiological mechanisms involved in the course of this endocrine disease. Recently, numerous authors have suggested that excess generation of highly reactive oxygen and nitrogen species is a key component in the development of complications invoked by hyperglycemia. Overproduction and/or insufficient removal of these reactive species result in vascular dysfunction, damage to cellular proteins, membrane lipids and nucleic acids, leading different research groups to search for biomarkers which would be capable of a proper and accurate measurement of the oxidative stress (OS) in diabetic patients, especially in the presence of chronic complications. In the face of this scenario, the present review briefly addresses the role of hyperglycemia in OS, considering basic mechanisms and their effects in diabetes mellitus, describes some of the more commonly used biomarkers of oxidative/nitrosative damage and includes selected examples of studies which evaluated OS biomarkers in patients with diabetes, pointing to the relevance of such biological components in general oxidative stress status of diabetes mellitus carriers.

## 1. Introduction

Diabetes mellitus (DM) is one of the most frequent chronic diseases worldwide, being among the top five main causes of death in developed countries. This endocrine disease is also becoming epidemic in developing countries [1]. The world prevalence of DM in 2010 was 6.6%, with an estimated number of 285 million carriers; by 2030, this number may reach 552 million carriers [2]. Type 2 diabetes mellitus (T2DM) is the most prevalent form of the disease, representing 90% to 95% of cases [3]. The evident increase in disease incidence and the higher frequency of chronic complications due to microvascular (e.g., nephropathy and retinopathy) and macrovascular (stroke, macrovascular coronary and peripheral artery diseases) alterations, as well as the difficulties for controlling it, makes DM a great challenge when considering health issues in the 21st century [4].

High morbidity and mortality rates are observed in DM carriers. For instance, the relative risk of death due to vascular complications is three-fold higher in patients with DM than in the remaining population [5], with cardiovascular diseases (CVD) being responsible for up to 80% of deaths in DM carriers [6]. In these patients, the risk of acute myocardial infarction (AMI) is similar to that observed in individuals with this previous condition [5].

The risk of developing CVD augments linearly with increased glycemia [7], favoring the emergence of cardiac disease, atherosclerosis, cataract, retinopathy, neuropathy and other complications present in poorly controlled DM [8–10]. Thus, there is a direct relationship between DM complications and chronic hyperglycemia (CH) [11,12]. In this context, oxidative stress (OS) has been proposed as a key mechanism reinforcing this relationship [13].

Different studies both in humans [14–16] and in animal models [17–21] have highlighted the complexity observed in multiple metabolic pathways involved in the determination of OS in DM. However, the whole picture in this scenario still remains to be determined. So, the present review, besides briefly addressing the role of hyperglycemia in OS, considering basic mechanisms and their effects in DM, aims to describe studies which evaluated OS biomarkers in patients with diabetes, pointing to the relevance of such biological components in general OS status of DM carriers.

## 2. Diabetes and Oxidative Stress

**Figure 1:**
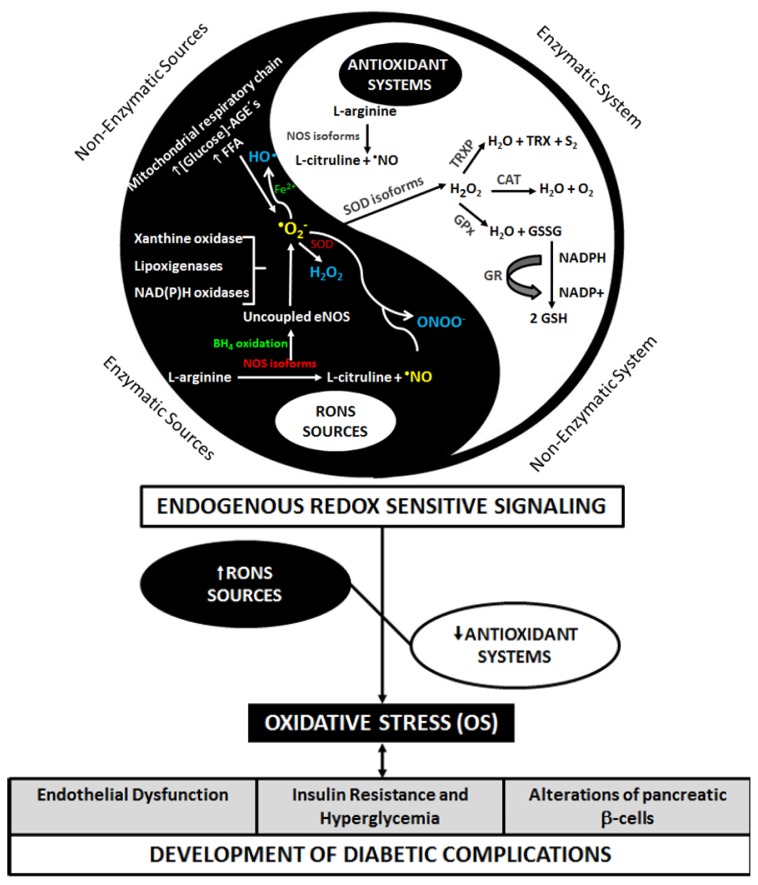
Schematic overview with some of the inter-related mechanisms involved in the regulation of oxidative balance. Physiologically, a tight control between antioxidants and oxidants is observed. In conditions of oxidative stress, however, an imbalance in favor of reactive oxygen and nitrogen species concentration occurs, leading to endothelial dysfunction, insulin resistance, and to alterations of pancreatic beta-cells. AGEs: advanced glycation end products; BH_4_: tetrahydrobiopterin; CAT: catalase; eNOS: endothelial nitric oxide synthase; FFA: free fatty acids; GPx: glutathione peroxidase; GR: glutathione reductase; GSH: reduced glutathione; GSSG: oxidized glutathione; H_2_O_2_: hydrogen peroxide; ^•^NO: nitric oxide; NOS: nitric oxide synthase; ^•^O_2_^−^: superoxide anion radical; ^•^OH: hydroxyl radical; ONOO^−^: peroxynitrite; RONS: reactive oxygen and nitrogen species; S_2_: sulfur; SOD: superoxide dismutase; TRX: thioredoxin; TRXP: thioredoxin peroxidase.

Oxidative stress was defined by Helmut Sies as the imbalance between antioxidants and prooxidants in favor of the latter, potentially leading to damage [22] (Figure 1). However, a broader concept was recently defined, including the disruption of redox signaling and control and/or molecular damage [23].

Jones [23] described the classical concept of OS in the cellular environment, then deepening this definition by highlighting that cells do not function as a uniform package, once every single organelle and cellular compartments present their own particular milieu. Thus, each of them would particularly include individual oxido-reduction processes often specific or more exuberant. Accordingly, OS would present itself compartmentalized in the nucleus, cell membrane or in the mitochondria, not being uniformly distributed along the whole cell structure. By further exploring this observation, he discusses the specificity either of reactive species or antioxidant mechanisms, emphasizing not only the participation of antioxidant components (such as extracellular superoxide dismutase, SOD and glutathione) but also the perturbation in the electron transfer among proteins or enzymes, thus leading to the formation of abnormal products (e.g., uncoupled endothelial nitric oxide synthase, eNOS). In fact, this broadened concept gives insight into the complexity of such regulatory redox mechanisms pointing to an infinite number of damage pathways, since diverse redox reactions in human biological systems exist. Thus, different cellular compartments seem to contribute differently to the maintenance of OS. In this line, mitochondria are described as playing an important role, representing a crucial regulator in the sustained metabolic imbalance observed in states of OS. The increase in reactive oxygen and nitrogen species (RONS) production in the intramitochondrial environment is deleterious to cellular functioning, once molecules like hydrogen peroxide (H_2_O_2_) and peroxynitrite (ONOO^−^) may cross mitochondria membranes and damage macromolecules in other cellular regions [24] (Figure 1).

**Figure 2:**
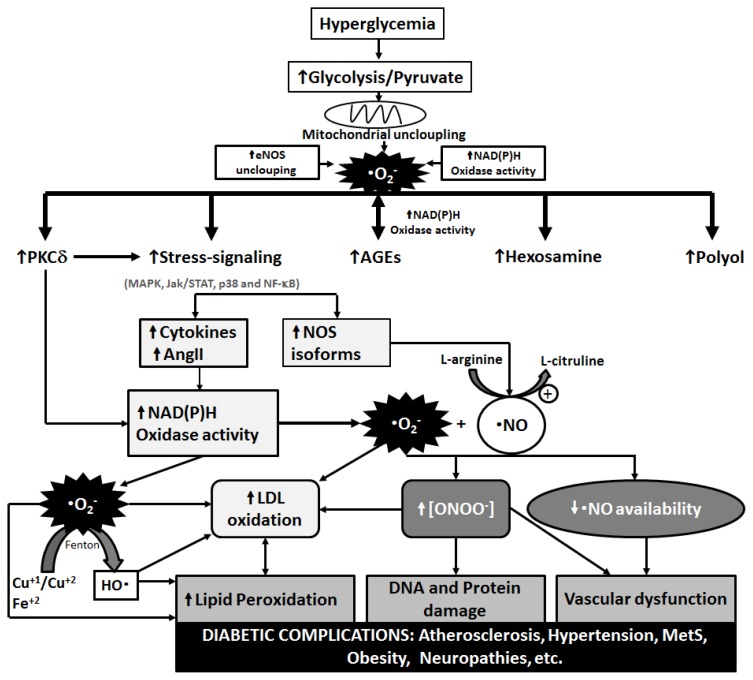
Participation of hyperglycemia in triggering the multiple oxidative stress pathways in the course of diabetes. AngII: angiotensin II; eNOS: endothelial nitric oxide synthase; Jac/STAT: janus kinase (Jac)-signal transducer and activator of transcription (STAT); LDL: low density lipoprotein cholesterol; MAPK: mitogen-activated protein kinase; MetS: metabolic syndrome; NF-κb: nuclear transcription factor κb; ^•^NO: nitric oxide; NOS: nitric oxide synthase; ^•^O_2_^−^: superoxide anion radical; ^•^OH: hydroxyl radical; ONOO^−^: peroxynitrite; PCKδ: protein kinase C δ. Adapted from Johansen *et al.*, 2005 [30].

In this context, the excessive production of superoxide anion radical (^•^O_2_^−^) in the mitochondrial electron transfer chain (ETC) [13] activates classical metabolic events evidenced in the course of DM, such as: increased polyol pathway activity [19]; increased formation of advanced glycation end products (AGEs) [25–27]; protein kinase C (PKC) [27,28] and nuclear transcription factor κb (NFκb) [29] activation; and increased hexosamine pathway flux [28]. In this scenario, hyperglycemia appears as a cornerstone, providing a favorable cellular environment for increased RONS production (Figure 2).

From the total consumed oxygen during oxidative phosphorylation, 0.4% to 4% are converted to ^•^O_2_^−^. Increased glucose levels overload cells with energy substrate, thereby augmenting the flux of electron donors (NADH and FADH_2_) to mitochondrial ETC. As a consequence, the voltage gradient of mitochondrial membrane reaches a critical threshold, blocking complex III and causing the return of electrons to coenzyme Q, which donates them to molecular oxygen, ultimately producing ^•^O_2_^−^ [13]. This usually occurs in complexes I and III of mitochondrial ETC [10]. In DM, however, complex II seems to play a more important role, as it produces a larger amount of ^•^O_2_^−^ [17]. Ten to 15% of oxygen molecules are not consumed in the mitochondrial respiration, being used by different enzymes in other cellular functions, such as synthesis of metabolites (through xanthine oxidase pathway), production of biological mediators (neurotransmitters and serotonin), and the elimination of exogenous compounds, via cytochrome P450 system, with the possible formation of reactive oxygen species (ROS) in these pathways [9].

The levels of RONS (Table 1) in an organism may still augment by increased NADPH oxidase activity—particularly involved in vascular OS [31], inflammatory cytokines, glycemia and free fatty acids (FFA) [32]. Oxidative phosphorylation, glucose autoxidation, increased lipoxygenase expression, changes in the regulation and expression of nitric oxide synthase (NOS) isoforms (endothelial NOS–eNOS, inducible NOS–iNOS, e neuronal NOS–nNOS) and ONOO^−^ production [10] are some of the mechanisms by which hyperglycemia can generate RONS (Figure 1).

A sustained increase in RONS and a decrease in endogenous antioxidant defenses contribute to the establishment and maintenance of OS, leading to endothelial dysfunction (ED), insulin resistance (IR), and to alterations of pancreatic beta-cells (Figure 1). These effects will lead to postprandial hyperglycemia and CH (and subsequently DM), which may increase the production of RONS [33], establishing a cyclic relationship between DM and OS, therefore triggering deleterious cellular processes. According to Halliwell and Gutteridge [9], it is not yet clear if OS leads to DM, but it is evident that DM promotes OS.

## 3. Metabolic Pathways of Diabetic Complications and the Role of OS

Oxidative damage may contribute to the development of complications linked to diabetes [15], being well accepted that RONS production induced by hyperglycemia represents the key event of such complications [17] (Figure 2).

An increased activity of polyol pathway, for instance, may augment cellular susceptibility to OS, since this will cause the consumption of NADPH, which besides serving as a cofactor of this pathway, is essential to regenerate glutathione (GSH), an important cellular antioxidant [20]. According to Lee and Chung [19], this pathway is the main source for RONS induced by hyperglycemia in retina. These authors also demonstrated that cataract derived from diabetes is proportional to the levels of aldose reductase (AR) and sorbitol in the retina of transgenic mice with AR overexpression. Usually, this enzyme reduces cellular toxic aldehyde into inactive alcohols. However, in the presence of high concentrations of intracellular glucose, AR reduces glucose to sorbitol, which is further converted to fructose through oxidation.

The increase in glucose flux through the polyol pathway may also explain diabetic neuropathy, as suggested by Ho *et al.* [20]. These authors investigated morphological abnormalities of peripheral nerves of diabetic mice with and without AR deficiency and verified a reduction of OS in the first group, suggesting the involvement of this pathway in the pathogenesis of acute diabetic neuropathy.

Another case is the AGEs, formed through non-enzymatic amino-carbonyl interactions between reducing sugars or oxidized lipids and proteins, aminophospholipids or nucleic acids [41]. The production of these compounds may lead to intracellular modifications of proteins, including those involved in the regulation of gene transcription [25,42]. Furthermore, studies in experimental animals [43] and humans [44,45] demonstrate the relationship between hyperglycemia and OS, with the formation of AGEs. Such compounds may spread to the outside of cells and modify molecules of the extracellular matrix, which may signal exchanges between the matrix and the cell, causing cellular dysfunction [25,26,46]. They are also capable of modifying circulating proteins in the blood that have receptors for AGEs (RAGE), activating them and inducing the production of inflammatory cytokines and growth factors in endothelial cells [21,26].

RAGE is a transmembrane protein that belongs to the cell surface immunoglobulin family [47] and is expressed in several cells and tissues, including endothelial cells [26,46]. The expression of the RAGE gene is low in tissues and vessels under normal physiological conditions, but increases in macrophages, monocytes, smooth muscle cells, endothelial cells and astrocytes upon an excess of AGEs [25].

Concerning its relation with OS, some authors suggest that AGEs may generate RONS [48,49]. In this regard, the binding of AGEs to RAGE, for instance, may promote OS through NADPH oxidase activation [47]. It may also activate NFκB, inducing the expression of pro-inflammatory cytokines, such as tumor necrosis factor (TNF) alpha and beta, interleukins (IL) 1α and 6, interferon gamma, endothelin-1 (EL-1), VCAM-1, selectin-1, tissue factor, thrombomodulin, tissue growth factor, and vascular endothelial growth factor (a specific mitogen of endothelial cells involved in angiogenesis and a mediator of late complications of diabetes) [25,50]. Moreover, NFκB activation may induce the expression of iNOS, increasing ^•^NO production [50]. ^•^NO will rapidly react with ^•^O_2_^−^ (*k* = 1.9 × 10^10^ × M^−1^ × s^−1^) upon an overproduction of the latter, with subsequent decrease of ^•^NO bioavailability and generation of ONOO^−^, a potentially toxic compound to endothelial cells [51], with all these events contributing to ED [47].

One of the explanations for the involvement of RAGE in inflammation is that the gene regions of NFκB and IL-6 are located at the RAGE promoter region, thus controlling the expression of this receptor in association with inflammatory responses [25]. NFκB plays an important role in mediating inflammatory immune responses and in apoptosis, regulating the expression of several genes, including VEGF and RAGE. VEGF expression increases in retina with the concomitant increase of AGE-RAGE interaction, giving rise to several cellular signaling cascades, including p44, p42, MAPKs and PKCs [29].

AGEs may also bind to other receptors, such as the macrophage scavenger receptor, p60, p90 and galectin-3. Furthermore, AGEs are described, besides markers, as mediators of late complications linked to diabetes and chronic vascular diseases, reinforcing their role in disease evolution and the complications and outcomes associated with it [26,29].

PKCs work as signaling molecules that regulate several cardiovascular functions, including vascular permeability, endothelial activation, myocardial contractility and growth factors [47]. An increase in diacylglycerol (DAG) synthesis, a cofactor for the classical forms beta, gamma and alpha [46] and the main physiological activator of this kinase, may be stimulated by hyperglycemia, affecting gene expression and associating this pathway to several processes present in diabetes complications [21,27,52]. PKC activation may also affect the production of oxidants and AGEs through the NADPH oxidase complex, causing alterations in cellular functioning [47] (Figure 2).

Besides the above mentioned signaling pathways activated by stress/inflammation (e.g., NFκB), other proteins such as c**-**Jun N**-**terminal kinases/kinases activated by stress (JNK/SAPK), and p38 kinase activated by mitogen (p38 MAPK) have been described (Figure 2). Thus, it is possible that the activation of NFκB is the initial event in response to RONS, followed by PKC activation, and generation of AGEs and sorbitol [53].

The activation of these pathways results in the upregulation of several genes that also cause cellular damage and have an important role in the etiology of late complications of diabetes [53]. In this case, OS figures as the connection between hyperglycemia and the physiopathology linked to diabetes [51], and may be the common pathway by which hyperglycemia and IR induce a decrease in insulin activity [54].

Of the four classical pathways involved in complications of diabetes, three (generation of AGEs, and activity of PKC and hexosamine) were blocked after inhibition of poly ADP ribose polymerase (PARP), suggesting that the inhibitors of this repair protein may prevent the development and progression of complications [28]. RONS produced during hyperglycemia induce breaks in nuclear DNA and activate PARP expression, which modifies glyceraldehyde-3-phosphate dehydrogenase (GAPDH) and decreases its activity, thus fostering complication pathways of diabetes [13,28].

Thus, both OS and the excessive glycation of proteins caused by hyperglycemia represent crucial components in the establishment of complications seen in diabetes. In this way, the physiopathology of diabetes may result from these two deleterious metabolic alterations that are activated by three main glycemic disorders: fasting hyperglycemia, postprandial hyperglycemia, and acute glucose fluctuations [55].

Accordingly, it appears that, despite the recognized physiological role of RONS, in the presence of exacerbated production of such species and/or in conditions of deficiency in antioxidant defenses to properly eliminate them, multidirectional damage to diverse cellular structures may occur. In this sense, the sum of these unfavorable conditions is already clearly identified in DM [10,34,56]. In fact, the better comprehension of the pathophysiological mechanisms linking diabetic complications and OS depends on the thorough observation of molecular pathways involved in the redox regulation, the reason why OS biomarkers are presented as critical tools for assessing the redox state in patients with DM.

## 4. Studies Assessing the OS in T2DM Carriers

An OS biomarker is defined as a biological molecule that can be measured and rightly reflects processes involving RONS, thus measuring OS in animals and humans [57]. As for the pathologies involving OS, such as DM, the optimal marker must accurately measure the different levels of this redox imbalance, allowing early diagnosis, identification of stage and progression of the considered disease, and evaluation of antioxidant therapies [58].

Although there is currently no optimal marker for OS linked to DM, the advances towards knowing the mechanisms involved in the DM-OS duo allowed the identification and utilization of biomarkers linked to its establishment and complications (Table 2), opening doors to the development of novel therapies.

The studies listed in Table 2 evaluated the relationship between T2DM and OS, especially diabetic complications, such as micro and macrovascular damage [59–61], neuropathies [40,62,63], atherosclerosis (with or without the presence of metabolic syndrome, MetS) [61], and obesity, with markers for oxidative damage and antioxidants [59]. The effect of insulin in attenuating OS by increasing antioxidant defenses was verified in a study conducted by Bravi *et al.* [64], both *in vitro* (incubation of erythrocytes with insulin) and *in vivo* (during hyperinsulinemic euglycemic clamp).

Besides verifying such associations, these studies also aimed to define the best biomarker of the binomial DM-OS (OS *vs.* DM or its complications, or associated diseases), or to verify the impact of antioxidant therapies. For instance, Piwowar *et al.* [59] performed the total radical-trapping antioxidant parameter (TRAP) test to evaluate protein damage through measurement of oxidative protein damage (OPD), and evaluated advanced oxidation protein products (AOPP). The authors concluded that AOPP might serve as efficient markers to estimate the level of protein damage mediated by oxidants in diabetic patients.

Similarly, Jaffar *et al.* [62] used the total antioxidant capacity (TAC) test and suggested that this approach can be useful to evaluate the impact of drugs or antioxidant treatments on delaying the onset of complications associated with OS. In this direction, the results obtained by Girona *et al.* [60] indicated that the levels of OxLDL/LDL, OxLDL/HDL and OxLDL-Ab are the best indicators for atherosclerosis in T2DM patients. With respect to antioxidant enzymes, Kimura *et al.* [61] suggested that extracellular superoxide dismutase (EC-SOD) levels could serve as a marker for vascular damage, possibly reflecting oxidative damage of vascular endothelium induced by hyperglycemia. Additionally, Ziegler *et al.* [40] cited polyneuropathy as a T2DM complication presenting severe OS. All these studies, through different means, point to the connection between OS biomarkers and glucose control, secondly highlighting the importance of an adequate treatment for DM, under penalty of establishing OS-mediated organic damage potentially irreversible. In this direction, recently, our group showed that T2DM carriers with inadequate glycemic control presented higher lipid peroxidation (LPO), when compared with non-T2DM and diabetic carriers with a good glycemic control [65].

In this regard, the work by Nojima *et al.* [80] is also relevant. Although these authors did not use any antioxidant, they treated patients (134 diabetic patients aged 30–74 years old) with regular moderate physical activity during twelve months. OS was then measured through the DNA damage marker 8-OHdG (8-hydroxydeoxyguanosine), and a decrease of this marker was observed after treatment, as well as a better controlled glycemic level associated with the decrease in OS. Although the authors recognized the study limitations due to sample size, their results corroborate others in that OS plays an important role in DM, being this imbalance measurable and could potentially be attenuated by proper treatment.

When considered the non-enzymatic mechanisms related to OS, a study by Ganesh *et al.* [66] showed increased LPO in T2DM carriers with iron deficiency compared to diabetic patients with proper concentrations of this mineral and to individuals without DM, pointing to the importance of iron metabolism in patients with the aforementioned endocrine disease.

In general, the works listed in Table 2 showed an increase in the concentrations of oxidative damage markers in T2DM carriers, and a decrease in antioxidant defenses when compared to controls, confirming the DM-OS relationship previously described in the literature. Moreover, they mention that OS may contribute to speed-up atherosclerosis, renal damage, neuropathy, cataract and retinopathy in diabetic patients, corroborating the above cited studies, once the evaluation of OS and complications of DM were positively correlated with an increase in OS makers and the presence of complications in T2DM.

Additionally, other authors have demonstrated increase in antioxidant defenses in T2DM carriers [61,65,67–69]. A study by Lima *et al.* [68], for example, sought to identify the relation of glucose control with zincemia and SOD activity, showing higher enzymatic activity in T2DM individuals compared to controls, as well as higher serum and erythrocyte zinc concentrations in the diabetic group. In line with these findings, Moussa [67], Bandeira *et al.* [65], and Soliman [69] found increased SOD activity and LPO in T2DM patients compared to controls, while Savu *et al.* [70] showed higher TAC and residual antioxidant activity in diabetic individuals, these observations associated with greater LPO.

Considering the studies presented herein, despite the fact that markers of LPO were the most evaluated ones (among 17 studies which assessed the occurrence of damage, 12 did so investigating LPO), some authors studied the oxidative damage to DNA [63,71–73] and proteins [59,72], verifying increased damage in T2DM carriers compared to individuals without the disease. In this context, an important consideration comes from the studies by Song *et al.* [73] and by Al-Aubaidy and Jenelek [71], as both revealed that, even in pre-diabetic states, DNA damage can already be identified. These findings evidence that diabetic complications seem to be preceded by harmful metabolic effects evoked even before the development of DM.

## 5. Main Biomarkers Evaluated in the Clinical Studies Presented

Among the 22 clinical studies cited in this review, 17 considered enzymatic and/or non-enzymatic antioxidants for evaluation, 8 of which (47%) found diminished defenses in T2DM carriers, with 8 (47%) observing increased antioxidant defenses. However, in all studies that assessed damage markers for macromolecules, levels of such markers were shown to be increased in diabetic individuals compared to non-diabetic ones. Taken together, these results suggest that the increase in antioxidant defenses may be related to an organic adaptive response in face of an environment favorable to the establishment of OS, the latter a feature of T2DM patients, finally evidencing the involvement of OS in the pathophysiology of T2DM. Nevertheless, considering the natural history of DM, it is possible to find a great diversity of this involvement along the course of the disease. Once this multifaceted scenario is presented, further studies are still needed, not only aiming to identify better biomarkers to be used in different phases of the clinical presentation of T2DM, but also to define specific values to be taken as reference in each phase of DM progression.

Nevertheless, if it is possible to achieve a consensus concerning the presence of OS in T2DM carriers, the same does not hold true when choosing appropriate biomarkers to be used clinically. In a review by Valko *et al.* [10], the main biomarkers of damage associated with DM were malondialdehyde (MDA) and F2-isoprostanes, reflecting the oxidative damage to lipids; reduced glutathione/oxidized glutathione (GSH/GSSG) ratio, S-glutathionylated proteins and 3-nitro-tyrosine (NO_2_-Tyr), as markers of oxidative damage to proteins; and AGEs, for assessing damage to lipids, proteins and nucleic acids. Considering these recommended biomarkers, among the studies which assessed oxidative damage, 47% evaluated MDA levels, with 11.77% focusing on F2-isoprostanes and 5.90%, on GSH/GSSG. Additionally, 11.77% investigated the markers 8-OHdG (for DNA damage) and AOPP (for protein damage). Taken together, these results reveal a tendency for assessing oxidative damage to lipids in the related studies, this fact especially observed by the greater frequency of MDA use as biomarker.

Another factor, which deserves to be addressed, is represented by the clinical features of T2DM, being numbered its development stages and chronic complications, as stated above, besides the glycemic control. In this regard, results from Bandeira *et al.* [65] suggest that the single fact of being diagnosed with diabetes is not a determinant factor for a considerable occurrence of oxidative damage. However, it is important to emphasize the need for understanding the way such damage is presented in each stage of the disease, since the initial steps of IR, through glucose intolerance (GI), until the establishment of CH. In this sense, Al-Aubaidy and Jelenek [71] detected changes in the damage marker 8-OHdG even in non-diabetic individuals presenting with glucose intolerance. Similarly, Song *et al.* [73], when using the comet assay, a method capable of detecting breaks in single-strand DNA, also identified higher DNA damage in patients with GI, with increased MDA concentrations only observed in T2DM carriers. Taken together, these findings suggest that changes in some biomarkers, as observed for 8-OHdG, may be more sensitive for detecting oxidative damage than others. In T2DM patients, the DNA damage was also assessed by Kasznick *et al.* [63] and Tabak *et al.* [72], both of them verifying increased damage in T2DM individuals, with or without established complications compared to controls. In this context, it is of great importance to remember that damage to DNA molecules leads to the activation of PARP and, consequently, decrease of GAPDH activity, whose function is the final glucose metabolism, implying the activation of different pathways involved in diabetic complications, according to the statement of some authors [13,28], as previously described in this review.

Considering the organic molecules, it is of notice that lipids are, probably, the most susceptible biomolecules, which may be attacked by free radicals. LPO is a cascade of events resulting from the action of RONS on polyunsaturated fatty acids present in cell membranes [81], directly damaging this cell component or indirectly evoking impairment in other structures, through the production of reactive aldehydes [82]. These alterations may cause changes in permeability, implying alterations in ion flux and other toxic substances, subsequently with DNA injury, oxidation of low density lipoprotein (LDL) particles, and cellular matrix damage, ultimately resulting in irreversible injury and then cell death [81].

Besides considering the aforementioned oxidative pathways, one cannot fail to mention the alterations in antioxidant defenses. This current review brings studies in which included individuals showed greater oxidative damage, even though with some antioxidant defenses increased [65,67,69,74]. Such observation points to the novel considerations concerning the OS already cited in this review [23], corroborating the complexity of the oxidative response, mainly in diseases affecting multiple organs and systems, as exemplified by T2DM.

In the context of antioxidant enzymes activity, SOD, for example, was shown to be increased in individuals with T2DM compared to controls in a broad range of studies [65,67–69,74]. Considering these variants, Kimura *et al.* [61] identified increased levels of EC-SOD in T2DM carriers, especially in the presence of micro and macrovascular complications. On the other hand, diminished SOD activity was also observed in such individuals [63,73,75,78]. As for glutathione peroxidase (GPx) and catalase (CAT) enzymes, Colak *et al.* [75] detected reduced activity for both in patients with T2DM presenting with complications, whereas Kasznick *et al.* [63] observed diminishment for GPx activity in T2DM carriers presenting with neuropathy, while Moemen *et al.* observed a decrease in the activity of GPx in diabetic patients with proliferative neuropathy (PDR) [74]. Complementing the variety of presentation for the described enzymes in diabetic individuals, Moussa [62] and Bandeira *et al.* [65], however, did not show significant changes for these enzymatic activities in T2DM patients. Besides these diverse forms of enzymatic response, which seem to depend on the degree of oxidative damage, these enzymes appear rather interrelated, since physiologically, SOD exerts its function by dismuting ^•^O_2_^−^ into H_2_O_2_. Following this process, GPx and CAT are responsible for breaking H_2_O_2_ into water and molecular oxygen [9], representing well known antioxidant enzymatic defenses (Figure 1). Finally, still considering the antioxidant defenses, some authors assessed the TAC, with results showing reduction of these components in T2DM carriers [62,73,75]. These observations, however, were not shown to be a consensus, as other descriptions in the literature [70] point to the increase in such defenses when considered T2DM patients.

Based on the facts reported herein, oxidative damage may result not only from increase in reactive species production, but also from cellular failure in effectively counteracting the mechanisms responsible for amplifying such damage, either through enzymatic or non-enzymatic systems. These events could reflect a decrease in endogenous antioxidants, as well as a depletion of dietary antioxidants and other essential components (e.g., copper, zinc, iron, magnesium), iron overload and/or increased production of reactive species (as observed in conditions of exposure to high oxygen concentrations), presence of toxins which produce such species, or the excessive activation of natural producing systems of reactive species [9]. In face of so many OS patterns in the course of DM, this scenario seems to indicate that the oxidative response is possibly related to the stage of disease development, the degree of glycemic control, the presence of chronic complications and comorbidities (such as obesity), besides genetic factors and lifestyle.

## 6. Conclusion

The cellular and molecular mechanisms involved in OS presented in this review are crucial for comprehending the damage caused by DM, considering OS as a key event in the development of its complications. When this relationship is better understood, proper therapies may be implemented for carriers. This may involve not only the right choice of drugs, but also—and even more importantly—the identification of patients at higher risk for developing complications, in this way allowing early clinical interventions to improve the patient’s health and aiding on lowering the costs for DM treatments.

Paradoxically, besides the promising data found in several publications concerning the relationship between DM and OS, studies involving the use of antioxidants present conflicting results [30,58], indicating that additional studies are necessary to clarify the pathways involved in this metabolic inter-relationship.

In summary, considering the multiple deleterious effects of an impaired glycemic metabolism, the search for a better understanding of the mechanisms responsible for the cellular damage observed in hyperglycemia, hyperinsulinemia and IR has become essential. The identification of common factors among these mechanisms represents one of the most important alternatives to dismantling the cyclic structure that sustains the chronic lesion patterns in diabetic patients.

## Figures and Tables

**Table 1:** Main reactive oxygen and nitrogen species (RONS) with known biological relevance.

Reactive Species	Reactivity	Source	Activity	Reference
^•^O_2_^−^	Low	ETC, phagocytic cells, autoxidation reactions (myoglobin, hemoglobin, catecholamines), enzymatic reactions (xanthine oxidase and NADPH oxidase), smoking	Vascular regulation; response to OS and maintenance of redox homeostasis	[34–36]
H_2_O_2_	Low	Mitochondrial matrix, ^•^O_2_^−^dismutation by SOD, xanthine dehydrogenase, smoking	May generate ^•^OH radical in the presence of transition metals, inactivate enzymes through oxidation of essential -SH groups	[10,37,38]
^•^OH	High	Water radiolysis, reaction of ^•^O_2_^−^ with H_2_O_2_ (Haber-Weiss) and in the presence of transition metals (Fenton), water ozonation and ONOO^−^ dissociation, smoking	May damage DNA, proteins, lipids and carbohydrates	[9,10,37]
O_2_^1^	High	Phagocytes, light induction, radiation, reactions catalyzed by peroxidases, smoking and others	Direct DNA damage, protein damage, initiates lipid peroxidation and produces alcoxyl and peroxyl radicals	[9,39]
^•^NO	High	NOS enzymes, smoking	Endothelium-derived relaxing factor. Reacts with other radical species, forming RNS that may modify DNA, proteins and lipids. Inhibits cytochrome P450 and some mitochondrial enzymes (ubiquinone oxidoreductase, oxidoreductase succinate and aconitase)	[34,40]
ONOO^−^	High	^•^O_2_^−^ reaction with ^−^NO, smoking	Lipid oxidation and nitration, DNA breaks	[9,10]
HClO	High	Reaction of H_2_O_2_ with Cl^−^ ions (catalyzed by myeloperoxidase)	Acts as a cellular defense against bacteria; may generate chlorinated amines that are strong oxidants	[9]

Notes: ^•^O_2_^−^: superoxide anion radical; H_2_O_2_: hydrogen peroxide; ^•^OH: hydroxyl radical; O_2_^1^: singlet oxygen; RNS: reactive nitrogen species; ^•^NO: nitric oxide; ONOO^−^: peroxynitrite; HClO: hypochlorous acid; -SH: thiol groups; ETC: electron transport chain; SOD: superoxide dismutase; Cl^−^: chloride; NOS: nitric oxide synthase.

**Table 2:** Case-control studies on OS in DM carriers.

Study sample	Biomarkers	Results with significant differences	Reference
T2DM = 94, C = 36	TRAP, OPD (SH, CO, NH_2_), AOPP	↓ TRAP and ↓SH; ↑ CO, ↑NH_2_, ↑ AOPP; ↑ AOPP in diabetic patients with macroangiopathy compared with those presenting microangiopathy; ↑ AOPP progressive with BMI	[59]
DA(+) = 73; DA(−) = 93	Vit E OxLDL/LDL OxLDL/HDL OxLDL/Ab	DA(+) *vs.* DA(−): ↑ OxLDL/LDL, OxLDL/HDL and OxLDL/Ab; ↑ OxLDL/LDL, OxLDL/HDL in the presence of MetS	[60]
T2DM = 222; C = 75	EC-SOD	↑ EC-SOD; Positive correlation between EC-SOD levels and severity of micro and macrovascular complications	[61]
T2DM PN(−)/CAN(−) = 62; T2DM PN(+)/CAN(−) = 105; TDM PN(+)/CAN(+) = 22; C = 85	8isoPGF2α (antioxidant capacity – ^•^O_2_^−^); (antioxidant capacity –ONOO^−^); Vit E/Lip; Vit C	PN(+)/CAN(−) *vs.* C: ↑ 8isoPGF 2α, ^•^O_2_^−^; ↓ Vit E/Lip, Vit C and ONOO^−^; PN(+)/CAN(+) *vs.* C:↑ ^•^O_2_^−^; ↓ Vit E/Lip and ONOO^−^; PN(+)/CAN(+) *vs.* PN(−)/CAN(−): ↑ ^•^O_2_^−^; PN(+)/CAN(−) *vs.* PN(−)/CAN(−): ↑ ^•^O_2_^−^; ↓ Vit E/Lip, Vit C	[40]
T1DM = 61; T2DM = 124; C = 70	TAC	↓ TAC in the presence of PN and/or CAN	[62]
T2DM(+)DSP N = 16; T2DM(−) DSPN = 16; C = 19	CAT, SOD, TAS, ^•^NO, DNA oxidative damage (Comet assay, endonuclease assay)	T2DM(+)DSPN *vs.* C: ↓ SOD, ↓ GPx, ↓ ^•^NO, ↑ DNA damage; T2DM(−)DSPN *vs.* C: ↓ ^•^NO, ↑ DNA damage; T2DM(+)DSPN *vs.* T2DM(−)DSPN: ↑ DNA damage	[63]
T2DM = 10 (Incubation of erythrocytes with insulin); T2DM = 14 (During clamp); C = 24	GSH/GSSG; TBARS; During hyperinsulinemic euglycemic clamp; GSH/GSSG; Erythrocytes incubation	D *vs.* C: ↑ GSH/GSSG; ↑ GSH/GSSG—after 2 hours of insulin incubation; ↑ GSH/GSSG—crescent after 60 and 120 min (during clamp); TBARS—no alteration All *vs.*	[64]
T2DM = 55; T2DM(−)LPO = 29; 2DM(+)LPO = 25; Pre-DM = 9;C = 29	SOD, CAT, GPx, uric acid, SH, CER, TRF, TBARS	T2DM *vs.* Pre-DM and C: ↑ SOD, ↑TBARS; T2DM and Pre-DM *vs.* C: ↑TRF; T2DM(+)LPO and Pre-DM *vs.* C: ↑TRF; T2DM(+)LPO *vs.* Pre-DM and C: ↑SOD; T2DM(+)LPO *vs.* T2DM(−)LPO, Pre-DM and C: ↑ FG, ↑ HbA1c	[65]
T2DM(+)Iron. def = 30; T2DM(−)Iron.def = 30; C = 30	MDA, uric acid	T2DM(+)Iron.def *vs.* T2DM(−)Iron.def: ↑MDA, ↓ uric acid; T2DM(+)Iron.def *vs.* C: ↑ MDA, ↓ uric acid	[66]
T1DM= 95; T2DM= 30; C= 20	GSH, GSH-red, GP*_x_*, SOD, MDA	T2DM *vs.* C: ↑ SOD, ↑ MDA; T1DM *vs.* C: ↑ SOD, ↑ MDA	[67]
T2DM= 36; C= 37	SOD, serum and erythrocyte Zn	↑ SOD, ↑ Zn	[68]
T2DM = 80; C = 80	MDA, GSH, SOD	↑ MDA, ↑ SOD, ↓ GSH	[69]
T2DM = 15; C = 18	TAC, residual antioxidant activity, MDA, albumin, uric acid	↑ TAC, ↑ residual antioxidant activity, ↑ MDA, ↑ acid uric	[70]
T2DM = 35; Pre-DM = 8; C = 119	8-OHdG	T2DM *vs.* Pre-DM and C: ↑ 8-OHdG; Pre-DM *vs.* C: ↑ 8-OHdG	[71]
T2DM = 69; T2DM(−)C = 20; T2DM(+)C = 49 C = 20	HEL, AOPP, 8-OHdG, 15- F2t-IsoP, PON 1	T2DM *vs.* C: ↑ AOPP, ↑ 8-OHdG, ↑ 15-F2t-IsoP, ↓ PON 1; T2DM(+)C *vs.* T2DM(−)C: ↑ AOPP, ↑ 8-OHdG, ↓ PON 1	[72]
IGR = 16; T2DMrecent = 34; C = 27	GSH, MDA, SOD, TAC, comet assay	T2DMrecent *vs.* IGR and C: ↑MDA, ↑ DNA damage, ↓ TAC; IGR *vs.* C: ↑ DNA damage; T2DMrecent and IGR *vs.* C: ↓ SOD	[73]
D = 35; NPDR = 29; PDR = 40; C = 32	MDA; NO*_x_*; SOD; GP*_x_*	D *vs.* C: ↑ MDA, ↑ NOx, ↑ SOD; NPDR *vs.* C: ↑ MDA, ↑ NOx; NPDR *vs.* D: ↑MDA, ↑ NOx, ↓ SOD, NPDR *vs.* PDR: ↑ MDA, ↑ NOx, ↓ SOD; PDR *vs.* C: ↑ MDA, ↑ NOx, ↓ SOD, ↓ GPx; PDR *vs.* D: ↑ MDA, ↑ NO*_x_*, ↓ SOD, ↓ GPx	[74]
DC(+) = 69; DC(−) = 48; C = 42	SOD, GPx, GR, TAS, Bilirubin, uric acid	DC(+) *vs.* C: ↓ SOD, GPx and GR, TAS, ↑ Bilirubin and uric acid; DC(+) *vs.* DC(−): ↓ SOD, GPx and GR, TAS	[75]
T2DM = 50; C = 21; T2DM(−)C = 29; T2DM(+)C = 21	CER, TRF, MDA	T2DM *vs.* C: ↑ CER, ↑ MDA, ↓ TRF; T2DM(+)C *vs.* C: ↑ CER, ↑ MDA, ↓ TRF; T2DM(+)C *vs.* T2DM(−)C: ↑ CER, ↑ MDA	[76]
T2DMrecent = 20; T2DM = 20; C = 20	Serum MDA, MDA after oxidation, erythrocyte MDA, total-SH, GSH, uric acid	T2DMrecent *vs.* C: ↑ Serum MDA, ↑ MDA after oxidation, ↑ erythrocyte MDA, ↓ GSH, ↓ total-SH; T2DM *vs.* C: ↑ Serum MDA, ↑ MDA after oxidation, ↑ erythrocyte MDA, ↓ GSH, ↓ total-SH; T2DM *vs.* T2DMrecent: ↑ MDA after oxidation, ↑ erythrocyte MDA	[77]
T2DM = 68; T1DM = 12; C = 30	TBARS	DM *vs.* C: ↑TBARS	[78]
T1DM = 12; C = 5	Plasma GSH, GSSG GSH/GSSG, and MDA	T1DM *vs.* C: ↑ GSSG, ↑ GSSH/GSH ↑ MDA	[79]

Notes: AOPP: advanced oxidation protein products; BMI: body mass index; C: control group; CAN: cardiovascular autonomic neuropathy; CAT: catalase; CER: ceruloplasmin; CO: carbonyl groups; D: diabetic patients without retinopathy; DA(+): diabetic patients with atherosclerosis; DA(−): diabetic patients without atherosclerosis; DC(+): diabetic patients with complications; DC(−): diabetic patients without complications; DM: diabetic individuals; EC-SOD: extracellular superoxide dismutase; FG: fasting glycemia; GPx: glutathione peroxidase; GR: glutathione reductase; GSH: reduced glutathione; GSH-red: glutathione reductase; GSSG: oxidized glutathione; HbA1c: glycated hemoglobin; HDL: high density lipoprotein; HEL: N-(hexanoyl)lysine; IGR: impaired glucose regulation; Iron-def: iron deficiency; MDA: malondialdehyde; MetS: metabolic syndrome; NH_2_: amine groups; ^•^NO: nitric oxide; NO*_x_*: nitrite + nitrate; NPDR: diabetic patients with nonproliferative retinopathy; ^•^O_2_^−^: superoxide anion radical; ONOO^−^: peroxynitrite; OPD: oxidative protein damage; OxLDL: oxidized low density lipoprotein; OxLDL/Ab: anti-OxLDL antibodies; PDR: diabetic patients with proliferative retinopathy; PN: polyneuropathy; PN(−)/CAN(−): lack of PN and CAN; PN(+)/CAN(−): presence of PN and lack of CAN; PN(+)/CAN(+): presence of PN and CAN; PON1: paraoxonase 1; Pre-DM: pre-diabetic patients; SH: thiol groups; SOD: superoxide dismutase; TAC: total antioxidant capacity; TAS: total antioxidant status; TBARS: thiobarbituric acid reactive species; T1DM: type 1 diabetes mellitus carriers; T2DM: type 2 diabetes mellitus carriers; T2DM(+)DSPN: type 2 diabetes mellitus patients with distal symmetric polyneuropathy; T2DM(−)DSPN: type 2 diabetes mellitus patients without distal symmetric polyneuropathy; TRAP: total radical-trapping antioxidant parameter; TRF: transferrin; Vit C: vitamin C; Vit E/lip: vitamin E/lipid ratio; Zn: zinc; 8-OHdG: 8-hydroxydeoxyguanosine; 8isoPGF2α: plasma 8-iso-prostaglandin F_2α_; 15-F2t-IsoP: 15-F2t-iso-prostaglandin.
